# Supplementary material for: A spatial gradient of bacterial diversity in the human oral cavity shaped by salivary flow
Source: Nat Commun. 2018 Feb 14;9:681. doi: 10.1038/s41467-018-02900-1 (PMC5813034; doi:10.1038/s41467-018-02900-1)
Supplement: Supplementary file 1 — Supplementary Information [file 41467_2018_2900_MOESM1_ESM.pdf]

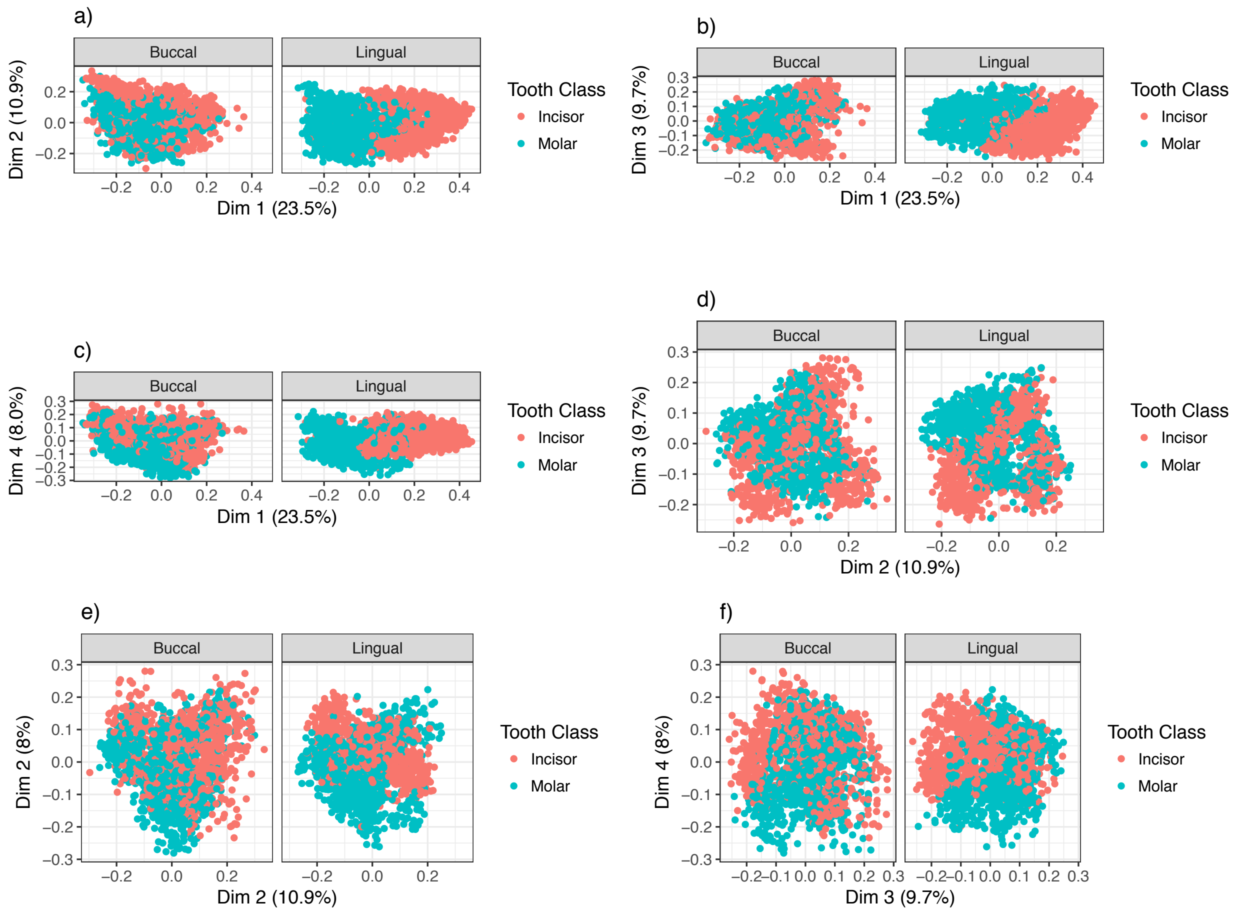

Supplementary Figure 1. Molar and incisor communities separate across axis 1 rather than across axis 2, axis 3 or axis 4. Each panel (a-f) represents a different pair-wise combination of ordination axes obtained from Principal Coordinates Analysis (PCoA) of Bray Curtis dissimilarity. In all cases, samples were colored by tooth class (molar vs. incisor) and samples were plotted separately based on tooth aspect (buccal/lingual). Incisor communities tended towards positive scores along axis 1 (x-axis) while molar communities tended towards negative scores regardless of whether the y-axis was the second (a), third (b) or fourth coordinate (c). On the other hand, separation of communities by tooth class across secondary axes was less distinctive (d-f).

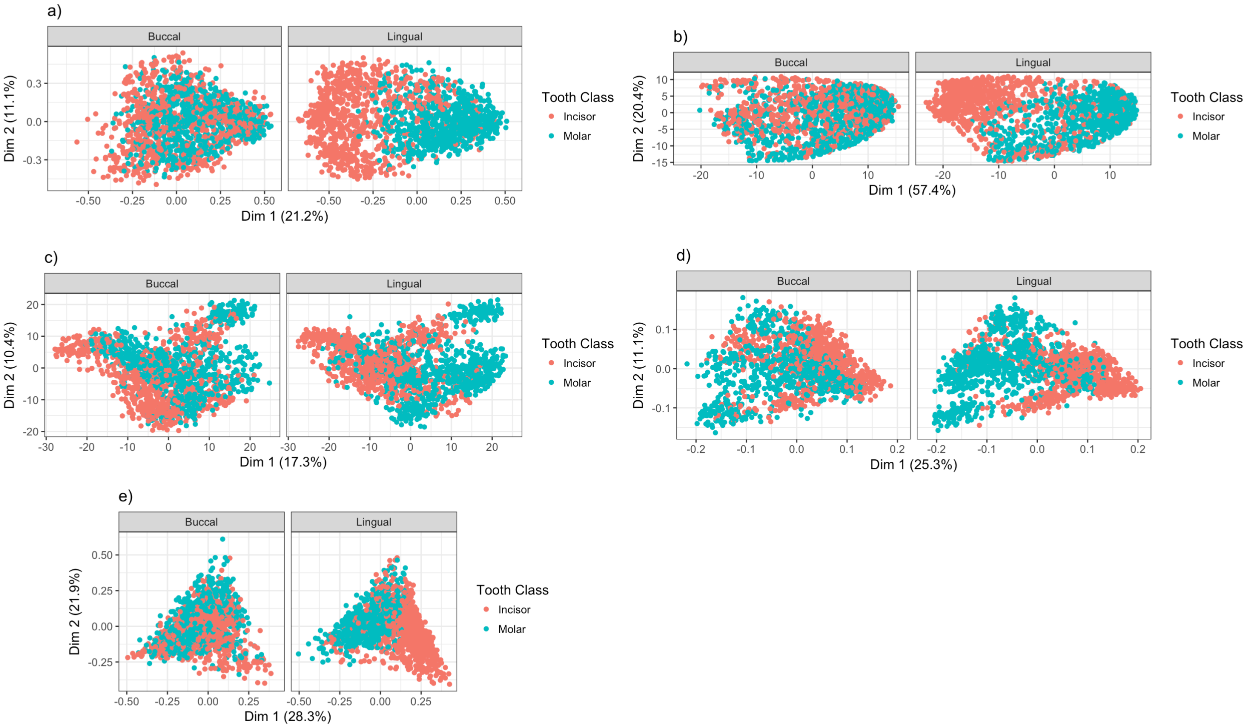

Supplementary Figure 2. Lingual molar and incisor communities are distinct irrespective of the transformation used prior to ordination. The index tooth data set was transformed using the a) Hellinger, b) Chord, or c) variance stabilizing transformation (VST) prior to ordination by PCoA. In addition, counts were converted d) to relative abundance or e) VST-transformed prior to computation of Bray Curtis dissimilarity. Molar and incisor samples separated across the first dimension when the a) Chord and b) Hellinger transformations were used prior to ordination of the between-sample Euclidean distance matrix as well as when a Bray Curtis dissimilarity matrix was computed on the d) relative abundance or e) VST-transformed data, but less so when c) the VST species profiles were ordinated directly. Regardless of transformation, the separation of molar and incisor samples tended to appear less striking for buccal than for lingual samples.

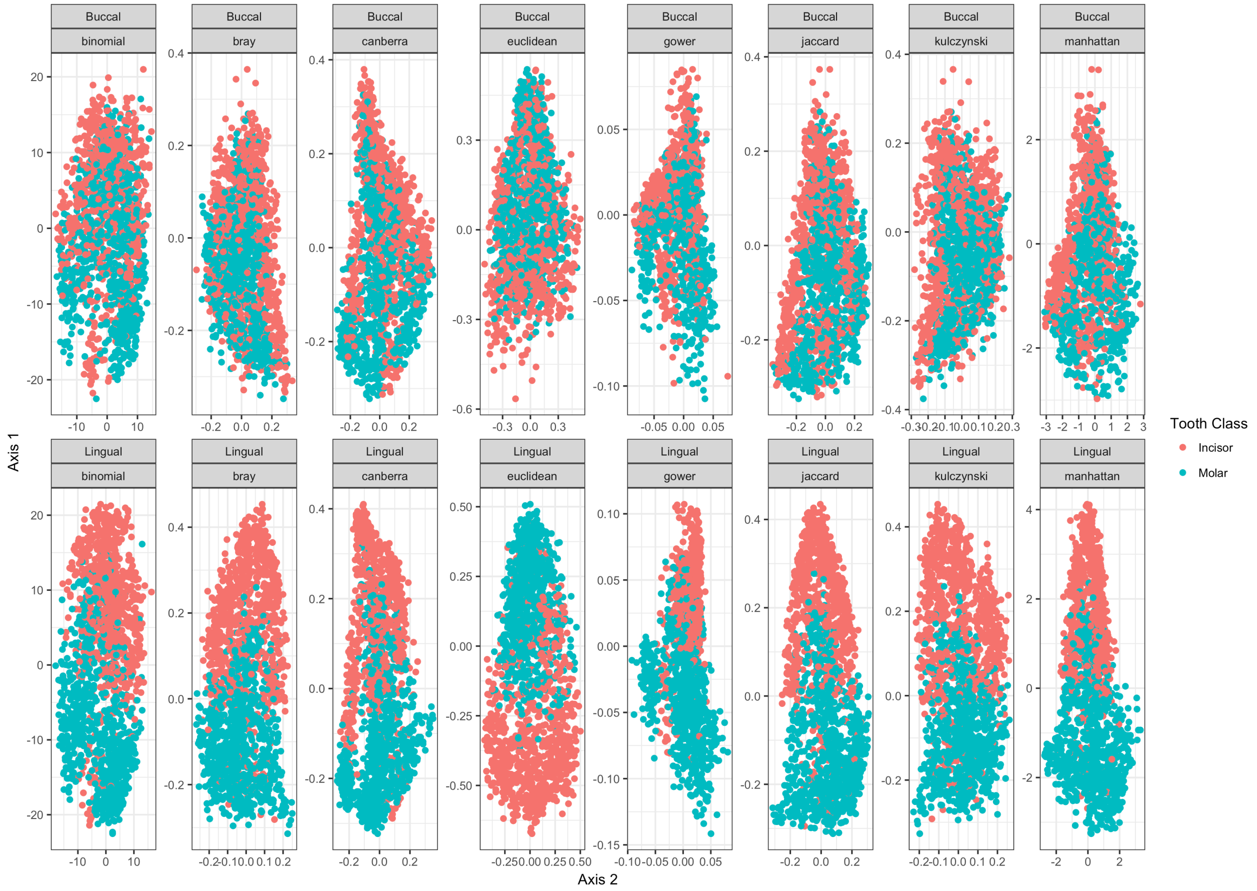

Supplementary Figure 3. Robustness of observation that molar and incisor communities are distinct. Several distance matrices (Binomial, Bray Curtis, Canberra, Euclidean, Gower, Jaccard, Kulczynski, Manhattan) were used as input for PCoA. Each panel depicts a distance metric and tooth aspect (buccal, lingual). Samples were projected onto the first (y-axis) and second (x-axis) coordinates and shaded by tooth class (molar vs. incisor). Irrespective of the distance metric, molar and incisor communities could be distinguished from one another along axis 1, particularly when communities were sampled from the lingual, rather than the buccal surfaces of teeth.

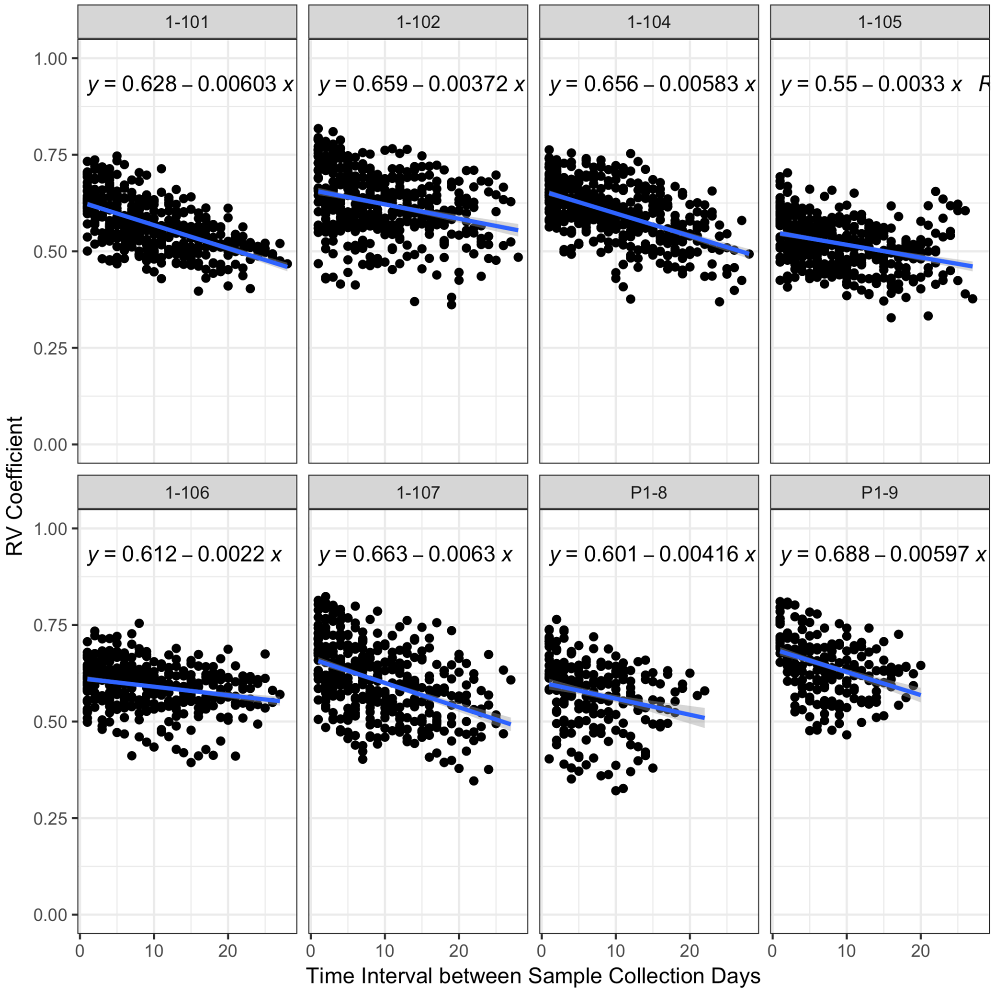

Supplementary Figure 4. Communities are relatively stable over time. The vectorial correlation (RV) coefficient was computed for each control subject and plotted as a function of the time interval separating pairwise sample collection days. Each panel represents a different subject. The blue lines represent a linear regression fit in which the time interval separating samples was used as the predictor of the RV coefficient while gray shading indicates 95% confidence intervals. The equations for each regression fit are displayed. The slope of the regression line was negative and small but significant for all subjects (Supplementary Data 2). These data suggest that community similarity gradually decays as the time interval separating samples increases over the course of 30 days.

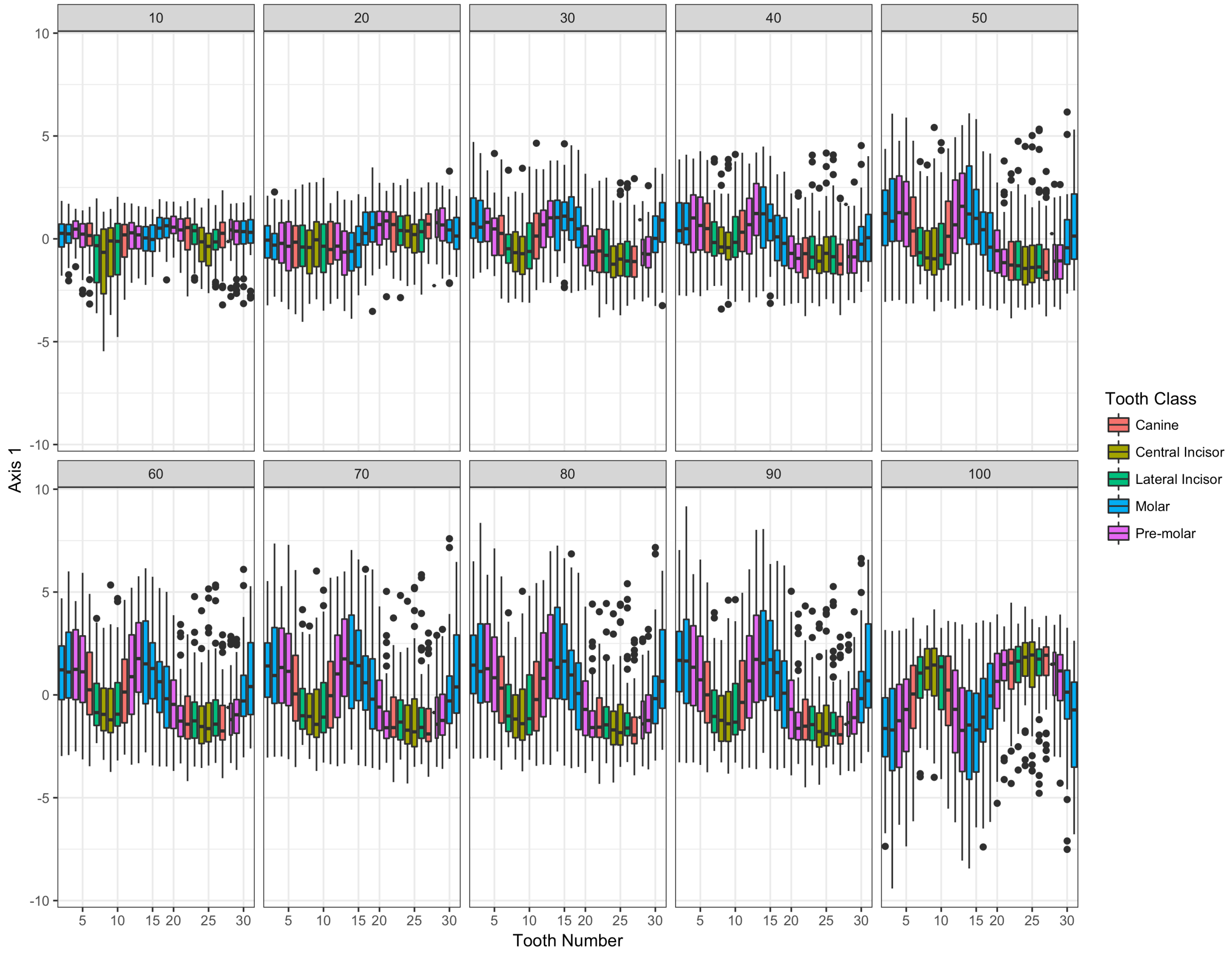

Supplementary Figure 5. Top 10-most abundant taxa appear to be generalists while less abundant taxa exhibit site specificity characteristic of the gradient. To evaluate the stability of the community gradient presented in Figure 2, we performed independent trend surface analyses on data subsets defined by different abundance thresholds (e.g., 10-, 20-, … , 100-most abundant taxa). Each panel represents an abundance threshold. Axis 1 scores (y-axis) are plotted against tooth number (x-axis), and boxplots are shaded by tooth class (canine, central incisor, lateral incisor, molar, pre-molar). Strikingly, the gradient could not be detected when only the 10-most abundant taxa were analyzed. The separation of tooth classes became increasingly apparent as more taxa were analyzed, indicating that the 10-most abundant taxa are generalists with respect to space while the less abundant taxa exhibit some degree of site specificity.

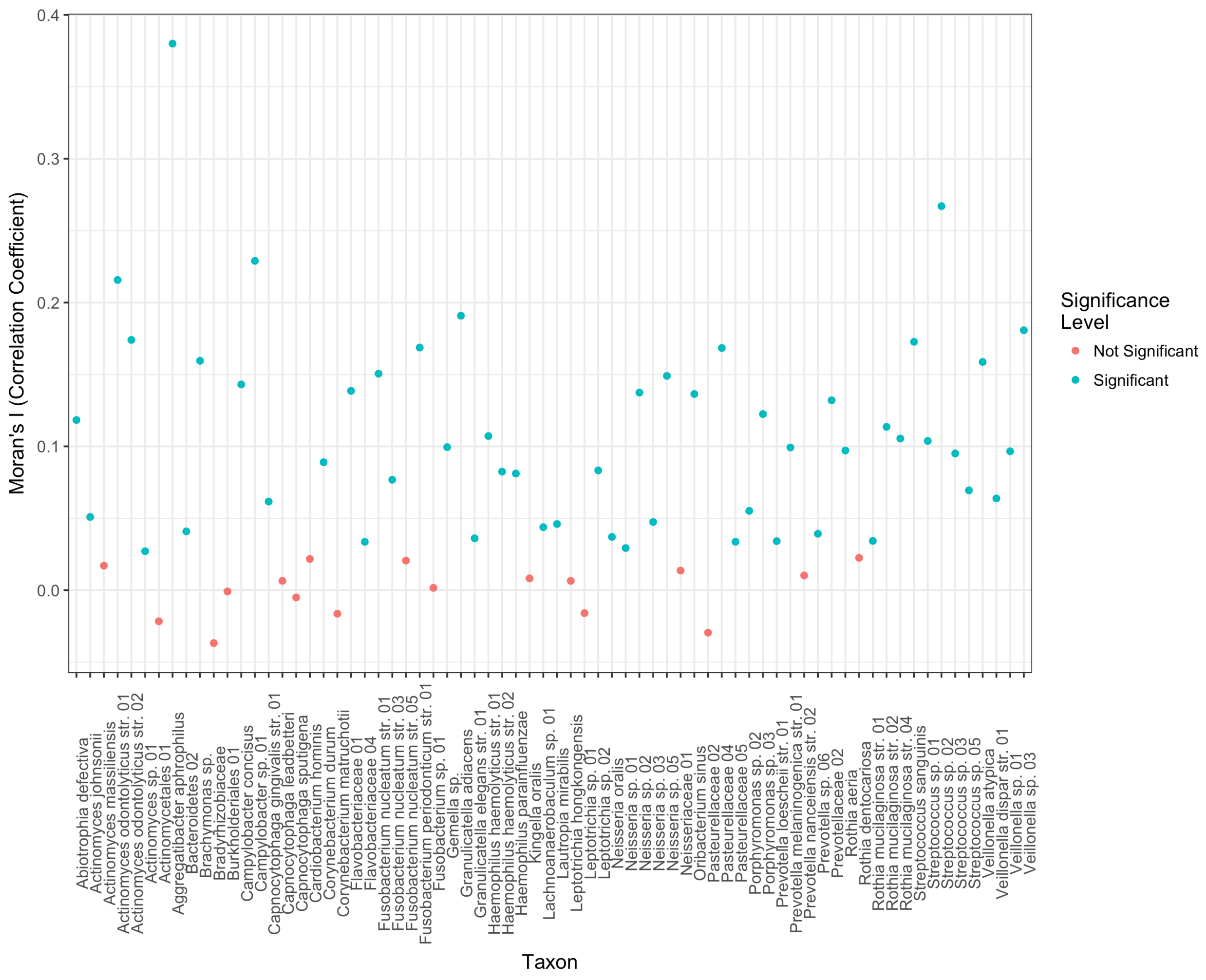

Supplementary Figure 6. Most (52 of 70) taxa exhibit a significant degree of spatial dependence that underlies the gradient (Figure 3). The Moran's *I* Coefficients (y-axis) are plotted for each of the 70-most abundant ASVs (x-axis). Each point represents a correlation coefficient and is colored according to whether the coefficient for the given taxon could be statistically distinguished from zero (i.e., **significant** or **not significant**). Taxa with the largest correlation coefficients included *Abiotrophia defectiva, Actinomyces odontolyticus str. 01, Fusobacterium periodonticum str. 01*, and a *Streptococcus sp.*

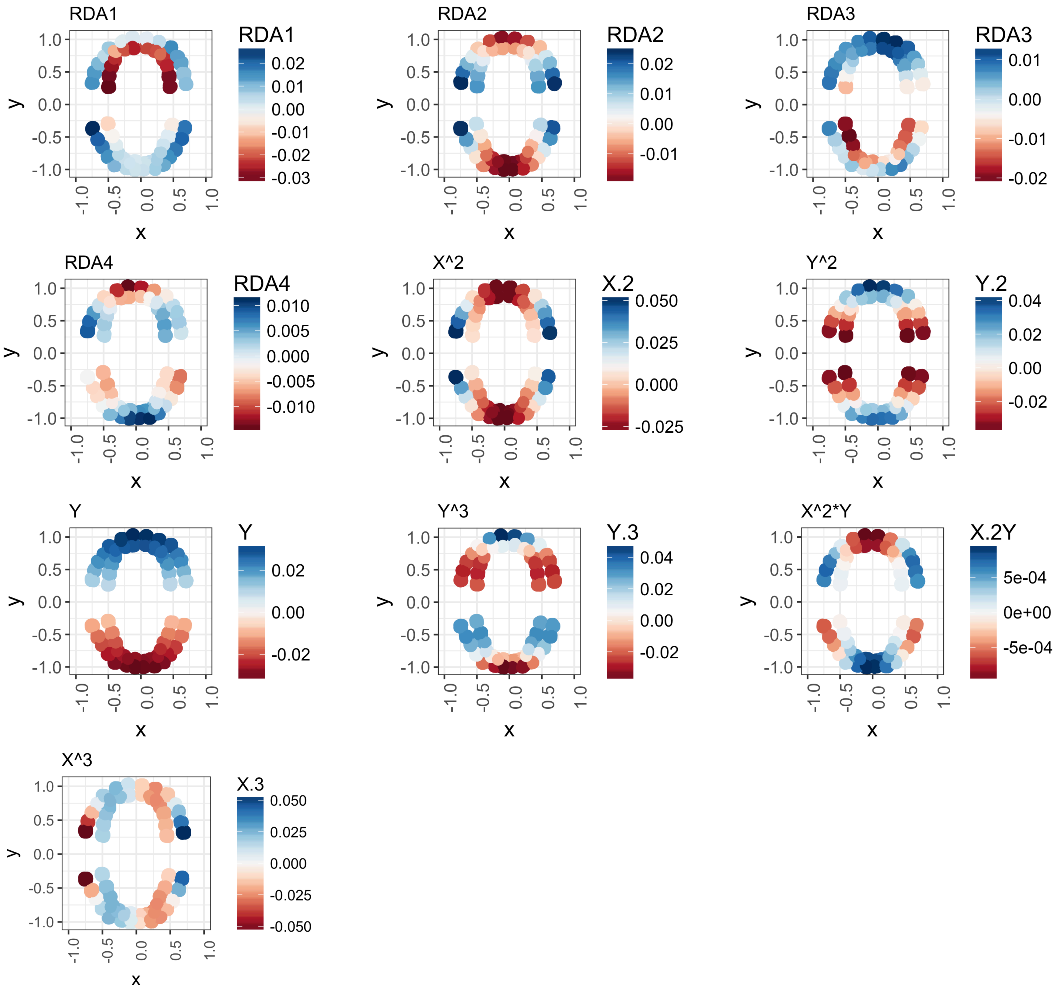

Supplementary Figure 7. Significant spatial structures identified by trend surface analysis. Redundancy analysis (RDA) axes that align with significant spatial structures are shown as heat-maps over the geographic coordinates of sites (x, y). RDA1 and RDA2 revealed interactions between the anterior-posterior gradient, jaw and tooth aspect. RDA3 primarily separated buccal and lingual surfaces in the lower jaw while RDA4 revealed an interaction between tooth aspect and position across the anterior-posterior dimension. In addition, 6 significant polynomial terms (X^2, Y^2, Y, Y^3, X^2Y, and X^3) are shown; these underlie the spatial structures observed by the RDA to drive significant spatial variation in microbial communities. For example, X^2 separates communities based on tooth position across the anterior-posterior dimension.

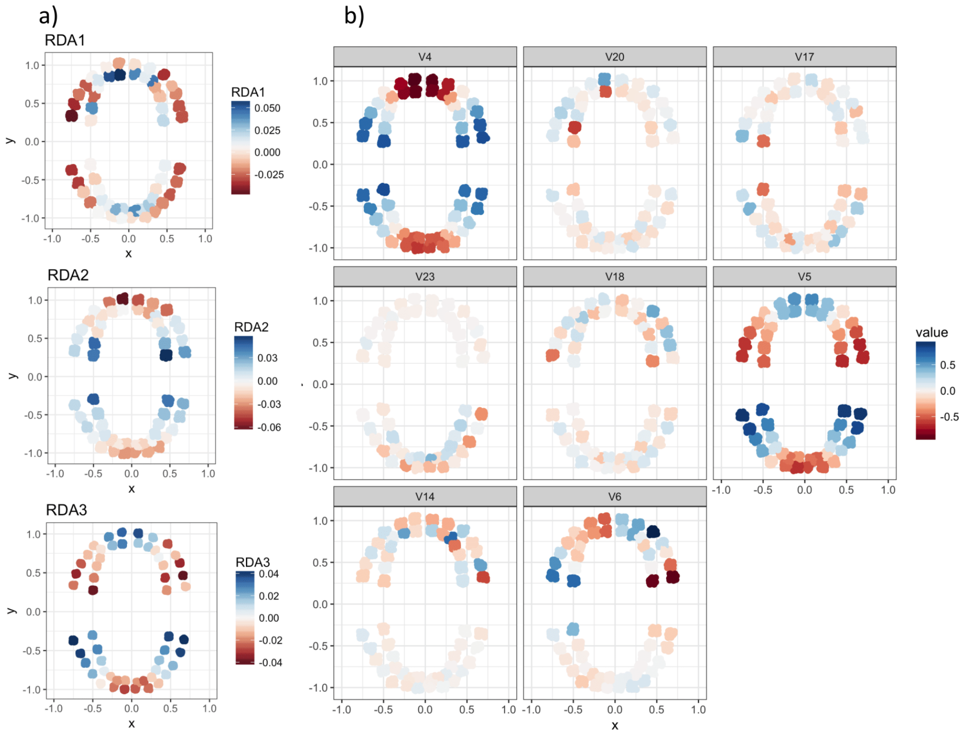

Supplementary Figure 8. Analysis of multiple spatial scales using principal components analysis of neighbor matrices (PCNM). a) RDA1 distinguished between the buccal and lingual tooth aspects; RDA2 corresponded to a 3-way interaction between the tooth, jaw, and tooth aspect; RDA3 differentiated anterior from posterior teeth in the upper and lower jaws. b) Of 8 significant PCNM variables, V4 and V5 aligned with broad scale spatial structures that likely correspond to RDA2 and RDA3. The remaining 6 PCNM variables corresponded to finer scale spatial patterns, highlighting differences between individual teeth (V6) or tooth aspects (V20, V17, V23, V18, V14). Taken together, these data suggested that teeth differ based on tooth aspect, jaw and position, highlighting again the importance of the anterior-posterior dimension.

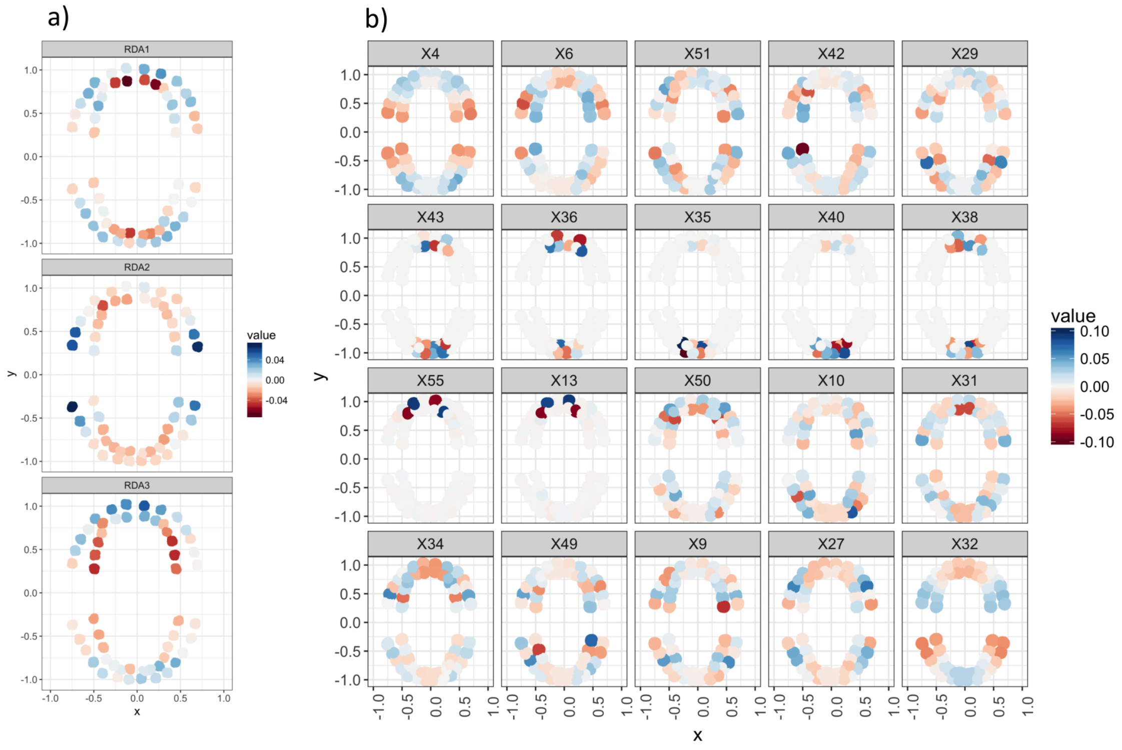

Supplementary Figure 9. Moran's Eigenvector Map Analysis (MEM) and model selection. a) RDA1 separated anterior from posterior sites in both jaws but only at lingual sites. RDA2 distinguished between anterior and posterior sites in the lower jaw. RDA3 represented an interaction between tooth aspect and variation across the anterior-posterior dimension. b) The significant MEM variables are shown in rank order by the percent variation explained. Of the 20 significant MEM variables, 13 (X4, X6, X9, X10, X13, X38, X40, X42, X43, X49, X50, X51, X55) corresponded to broad scale spatial patterns. The other 7 (X27, X29, X31, X32, X34, X35, X36) corresponded to fine scale spatial structures, which were difficult to interpret.

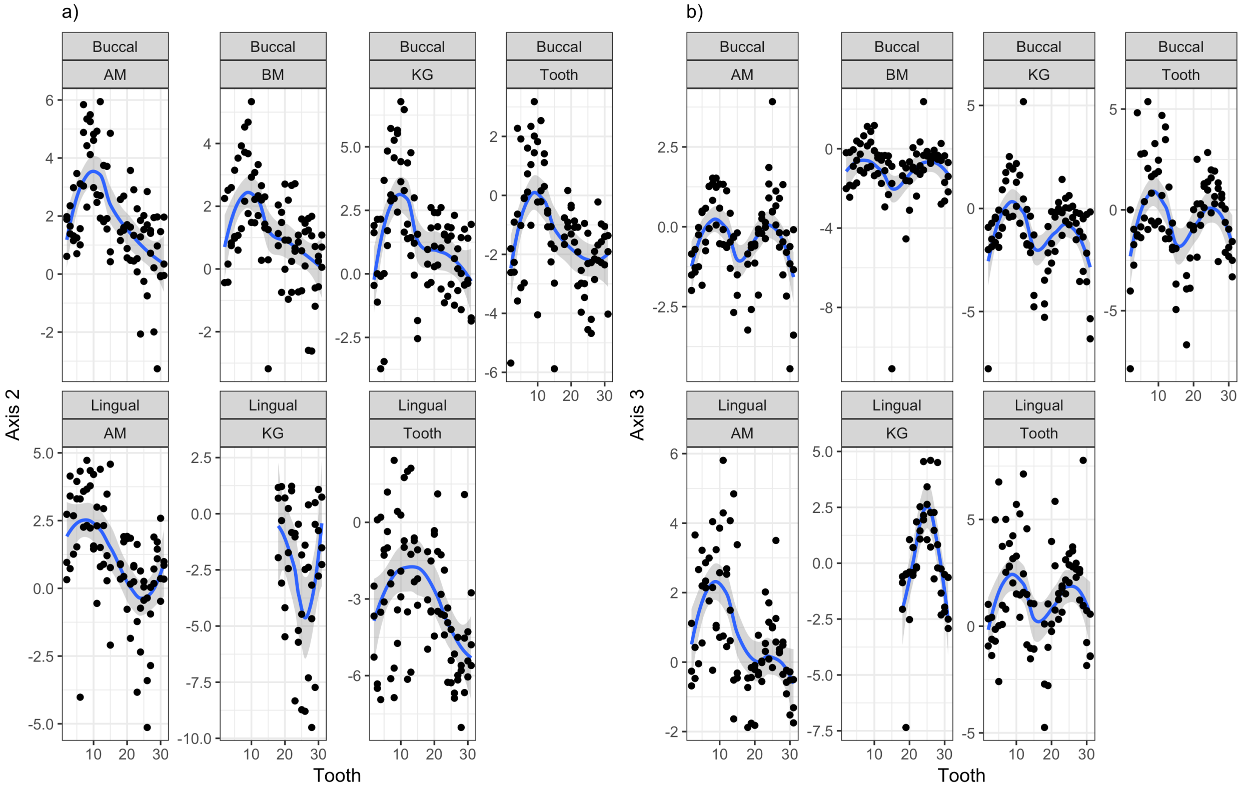

Supplementary Figure 10. Extended spatial analysis of the mucosal trend surface analysis. The a) second and b) third significant axes are plotted (y-axis) as a function of tooth number (x-axis). Each panel represents a tissue type (alveolar mucosa, buccal mucosa, keratinized gingiva, and supragingival plaque) and a tooth aspect (buccal, lingual). Each point represents a community sample, blue curves represent local regression fits (loess) while the gray shading indicates the 95% confidence interval surrounding each model fit. a) Axis 2 differentiated sites based on jaw (maxilla vs. mandible) while b) Axis 3 corresponded to the difference between the anterior and posterior mouth, similar to the first axis.

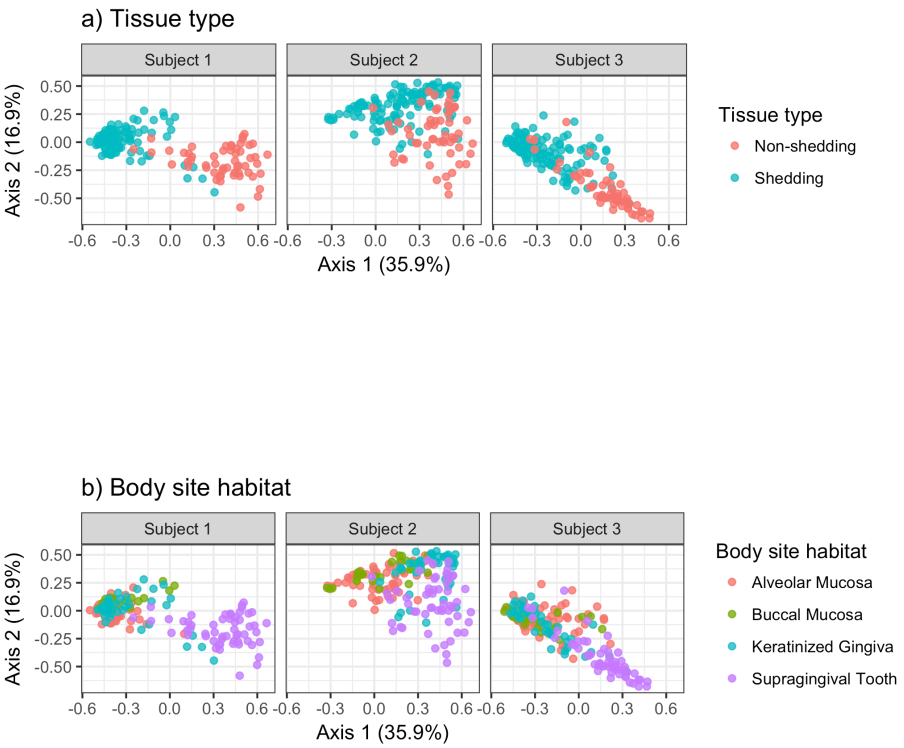

Supplementary Figure 11. Tissue type differentiates communities despite the shared gradient. Principal Coordinates Analysis (PCoA) of Bray Curtis dissimilarity was performed on the mucosal biogeography data set. Samples were projected onto the first (x-axis) and second (y-axis) coordinates. Each panel represents a different subject. Each point represents a sample, which is shaded based on a) whether the originating site was non-shedding (supragingival tooth) or shedding (alveolar mucosa, keratinized gingiva, buccal mucosa) or b) which body site habitat (alveolar mucosa, buccal mucosa, keratinized gingiva and supragingival tooth surfaces) was sampled. Community composition varied enough between tissue types and body site habitats for communities to segregate based on each factor despite the observation that they conformed to a common gradient separating molars and incisors.

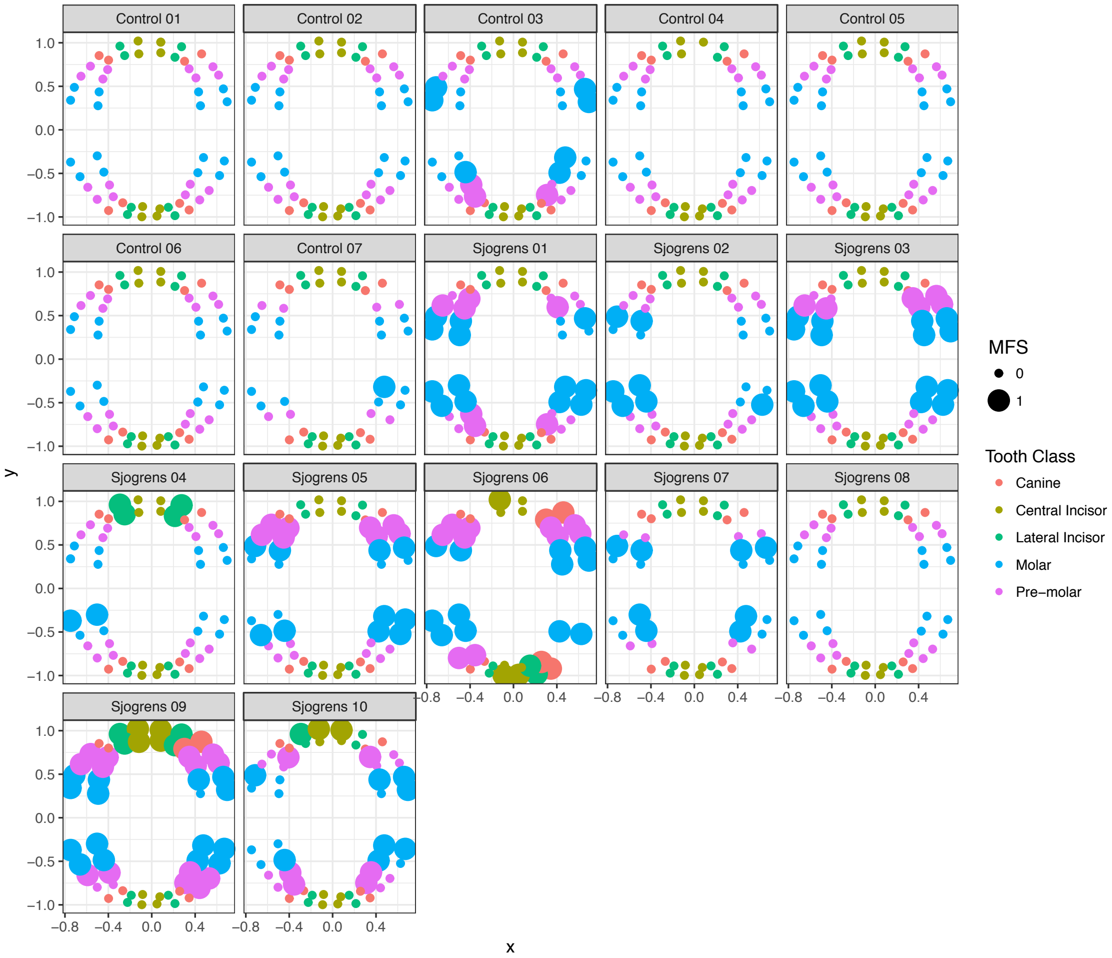

Supplementary Figure 12. Past history of caries. Each panel represents a subject. Each point represents an observation for a given tooth at either the buccal or lingual aspect. The size of each point indicates the presence or absence of missing, filled surfaces (MFS). Data are projected onto geographic coordinates (x, y) of sites. Colors represent tooth classes (canine, lateral incisor, central incisor, molar, premolar). Overall, individuals with SS (Sjögren's 01-Sjögrens 10) had a higher burden of MFS on front teeth (incisors, canines) compared to the controls (Control 01-Control 07).

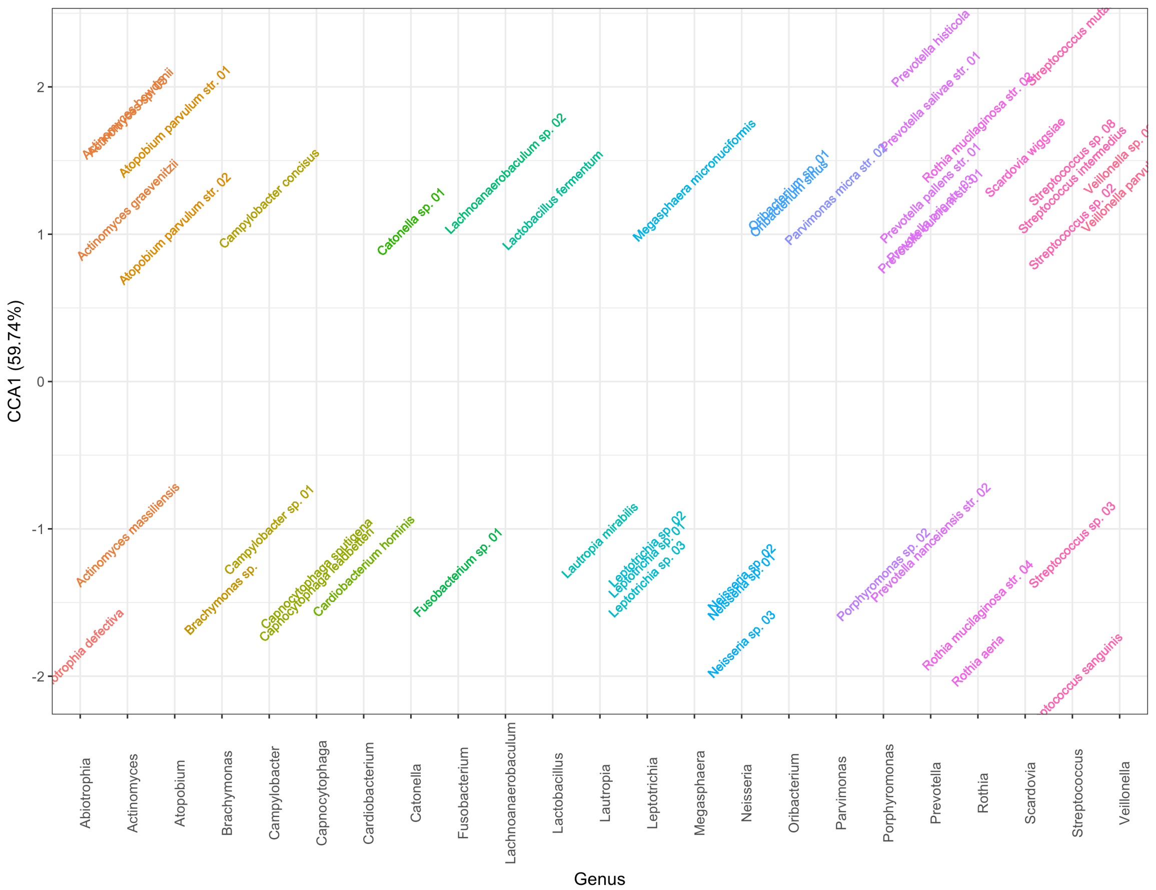

Supplementary Figure 13. Communities associated with low salivary flow are enriched in acid-loving, caries-associated taxa. Taxa are sorted by genus on the x-axis; Constrained Correspondence Analysis (CCA) axis 1 scores are displayed on the y-axis. Positive CCA1 scores, where low flow samples mapped, were associated with acid-loving, caries-associated taxa such as *Streptococcus mutans*, *S. intermedius, Lactobacillus fermentum*, *Scardovia wiggsiae*, *Oribacterium sinus*, and *Atopobium parvulum*[1], among others. Negative CCA1 scores, where control samples mapped, were associated with health-associated taxa including *S. sanguinis*, *Abiotrophia defectiva,* and *Capnocytophaga leadbetteri* [2], among others. These data suggest that individuals with low salivary flow are enriched in acid-loving or acid-producing, caries-associated bacteria.

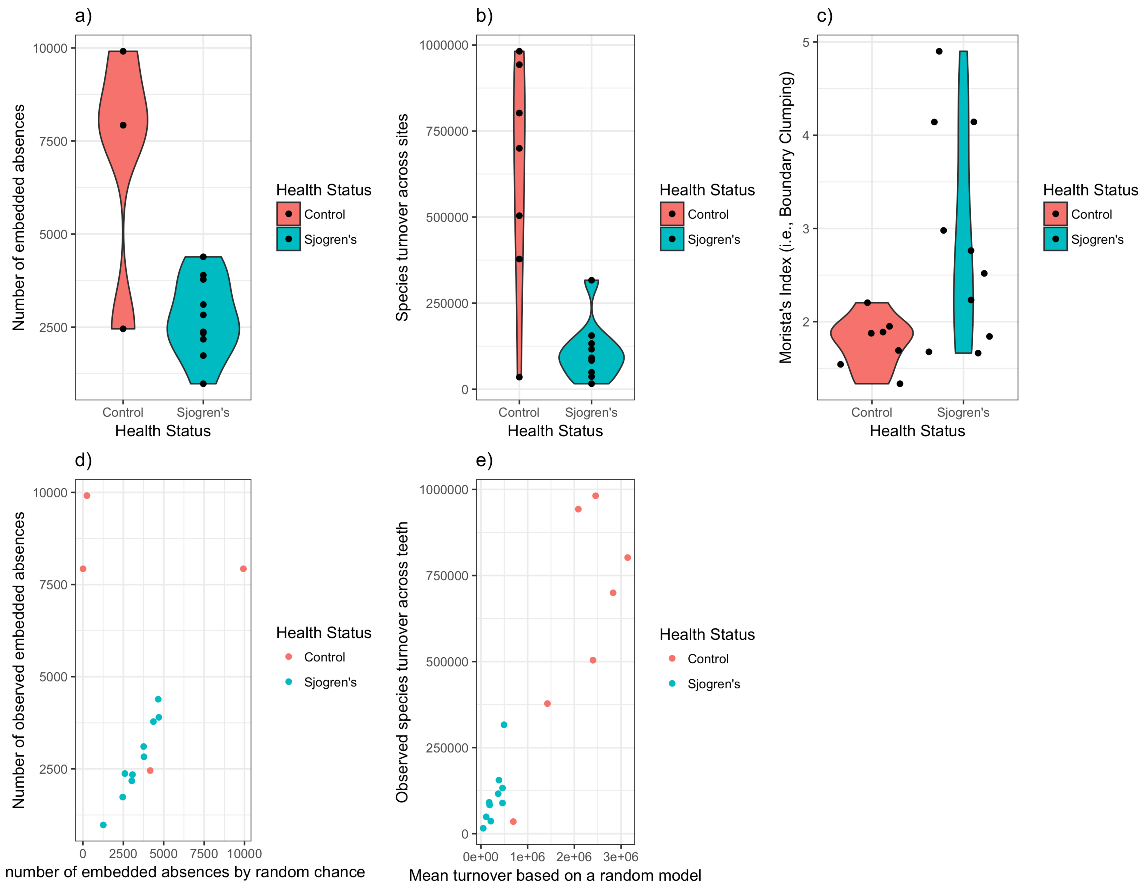

Supplementary Figure 14. Elements of Metacommunity Structure (EMS) Analysis. EMS analysis was performed on supragingival communities from healthy controls and from patients with low salivary flow due to Sjögren's Syndrome. a) Regardless of subject health status, coherence as measured by the number of embedded absences was significant. e) Moreover, the number of embedded absences was, in all cases, less than expected, as indicated by the difference between the simulated mean and the observed value. Turnover was b) significant and e) less than expected by random chance. c) The boundary clumping index exceeded 1, in all cases, suggesting that communities exhibit clumped species loss along the gradient. Taken together, these data suggest that communities are distributed along the anterior-posterior gradient in a manner characterized as a nested subset with clumped species loss.

PID	Project	Gender	Age	Racial Category	Sampling Period	Sampling Interval & Teeth Sampled
P2-1	Discovery	Male	ND	Asian	8 days	Every other day: 3, 8, 9, 14, 19, 24, 25, 30
P2-2	Discovery	Female	ND	Multiracial	8 days	Every other day: 3, 8, 9, 14, 19, 24, 25, 30
P1-1	Discovery	Female	ND	Asian	8 days	Daily: 3, 8, 9, 14, 19, 24, 25, 30
P1-2	Discovery	Female	45	White	8 days	Daily: 3, 8, 9, 14, 19, 24, 25, 30
P1-3	Discovery	Female	57	White	8 days	Daily: 3, 8, 9, 14, 19, 24, 25, 30
P1-4	Discovery	Male	59	White	8 days	Daily: 3, 8, 9, 14, 19, 24, 25, 30
P1-5	Discovery	Male	21	White	8 days	Daily: 3, 8, 9, 14, 19, 24, 25, 30
P1-6	Discovery	Female	46	White	8 days	Days 1 & 8: 2-15,18-31<br>Daily: 3, 8, 9, 14, 19, 24, 25, 30
P1-7	Discovery	Female	43	White	8 days	Days 1 & 8: 2-15,18-31<br>Daily: 3, 8, 9, 14, 19, 24, 25, 30
P1-8	Discovery	Female	35	White	29 days	Days 1 & 8: 2-15,18-31<br>Daily: 3, 8, 9, 14, 19, 24, 25, 30
P1-9	Discovery	Female	26	Asian	29 days	Days 1 & 8: 2-4,6-11, 13-15, 18-20, 22-27,29-31<br>Daily: 3, 8, 9, 14, 19, 24, 25, 30
4-101	Mucosa	Female	24	White	1 day	2-15,18-31<br>Also the oral mucosa
4-102	Mucosa	Female	46	White	1 day	2-15,18-31<br>Also the oral mucosa
4-103	Mucosa	Male		White	1 day	2-15,18-31<br>Also the oral mucosa
1-101	Validation	Female	24	White	29 days	2-15,18-31, once per week each week<br>3, 6, 8, 9, 11, 14, 19, 22, 24, 25, 27, 30, daily
1-102	Validation	Male	ND	White	29 days	2-15,18-31, once per week each week<br>3, 6, 8, 9, 11, 14, 19, 22, 24, 25, 27, 30, daily
1-103	Validation	Female	ND	Multiracial		
1-104	Validation	Female	23	Asian	29 days	2-15,18-31, once per week each week<br>3, 6, 8, 9, 11, 14, 19, 22, 24, 25, 27, 30, daily
1-105	Validation	Female	55	Asian		2-15,18-31, once per week each week<br>3, 6, 8, 9, 11, 14, 19, 22, 24, 25, 27, 30, daily
1-106	Validation	Female	28	Asian	29 days	2-15,18-31, once per week each week<br>3, 6, 8, 9, 11, 14, 19, 22, 24, 25, 27, 30, daily
1-107	Validation	White	22	Male	29 days	2-15,18-31, once per week each week<br>3, 6, 8, 9, 11, 14, 19, 22, 24, 25, 27, 30, daily
3-301	Validation	White	54	Female	1 day	2-15,18-31
3-302	Validation	Female	63	White	1 day	2-15,18-31
3-303	Validation	Female	59	Asian	1 day	2-15,18-31
3-304	Validation	Male	54	White	1 day	2-15,18-31
3-305	Validation	Female	53	Asian	1 day	2-15,18-31
3-306	Validation	Female	67	White	1 day	2-15,18-31
3-307	Validation	Female	70	White	1 day	2-15,18-31
3-308	Validation	Female	55	Hispanic or Latino	1 day	2-15,18-31
3-309	Validation	Female	ND	ND	1 day	2-15,18-31
3-310	Validation	Female	76	White	1 day	2-15,18-31

Supplementary Table 1. Characteristics of the human subjects enrolled in the discovery, validation and mucosa biogeography cohorts. Subjects were recruited into one of three projects. The project, gender, age, and racial identity of each human subject is shown (if known). In addition, we describe the sample collection protocol for each subject, including the sampling interval (whether daily or every other day) as well as the teeth that were sampled. Not determined (ND) indicates that data are not available.

Phylum	Abundance	Percent
Firmicutes	196601153	39.815
Proteobacteria	139356937	28.2182
Actinobacteria	108027071	21.8742
Bacteroidetes	34477486	6.9813
Fusobacteria	14858582	3.0087
SR1	340398	0.0689
Spirochaetes	121010	0.0245
Cyanobacteria/Chloroplast	52172	0.0106
Candidatus Saccharibacteria	10815	0.0022
Aquificae	3559	7.00E-04
Acidobacteria	2799	6.00E-04
Deinococcus-Thermus	1701	3.00E-04
Tenericutes	1296	3.00E-04

Supplementary Table 2. Phylum level description of observed taxa. A total of 13 phyla were observed in the validation data set (480 taxa, 7002 samples). The number of observed sequences for each phylum is displayed as the abundance value and the percent abundance for each taxon is displayed as the percent. The top 5 most abundant phyla (Firmicutes, Proteobacteria, Actinobacteria, Bacteroidetes and Fusobacteria) accounted for 99.89% of all reads.

Genus	Abundance	Percent	Number of ASVs
Streptococcus	143337443	31.6955	11
Haemophilus	64073059	14.1682	6
Rothia	61438005	13.5855	6
Neisseria	32952227	7.2866	10
Actinomyces	25749541	5.6939	17
Corynebacterium	19371708	4.2836	4
Veillonella	17758620	3.9269	10
Abiotrophia	11542724	2.5524	1
Gemella	11334234	2.5063	2
Prevotella	11301369	2.499	46

Supplementary Table 3. Genus level description of observed taxa in the validation data set. A total of 118 genera were observed in the validation dataset (480 taxa, 7002 samples). The number of observed sequences for each genus is displayed as the abundance value and the percent abundance is displayed as the percent. The number of ASVs per genus is also shown. The top 10 most abundant genera (*Streptococcus*, *Haemophilus*, *Rothia*, *Neisseria*, *Actinomyces*, *Corynebacterium*, *Veillonella*, *Abiotrophia*, *Gemella* and *Prevotella*) accounted for 90.87% of all reads. *Prevotella* was the most diverse genus with 46 different ASVs while *Abiotrophia* was the least diverse with only one ASV, corresponding to *Abiotrophia defectiva*.

Supplementary Notes

Supplementary Note 1. Robustness of the observation that the molars and incisors are different

Adonis

Since examination of the Principal Coordinates Analysis (PCoA) presented in Figure 1 suggested an interaction between tooth class and tooth aspect we sought to quantify the relative influence of each factor (Subject, Tooth Aspect, Tooth Class) on the structure of oral microbial communities using a permutational analysis of variance (Adonis), as discussed in the main manuscript. Here, we present the full Adonis table.

To contend with the non-independence of temporal replicates, we collapsed temporal replicates by summing across time within each individual before the analysis. This analysis revealed interpersonal differences contribute the most to the variation of bacterial communities ($F=35.7$, $R^2=0.469$, $p<0.05$). Tooth class explained ~9.4% of the total variation ($F=42.7$, $R^2=0.094$, $p<0.05$) while tooth aspect explained ~3.8% of the total variation ($F=17.1$, $R^2=0.038$, $p<0.05$). The interaction between subject and tooth class indicates personalized differences in community composition based on tooth class ($F=7.3$, $R^2=0.095$, $p < 0.05$) similar to the interaction between subject and tooth aspect ($F=3.3$, $R^2=0.043$, $p<0.05$). The interaction between tooth class and tooth aspect was also significant ($F=1.216$, $R^2=0.043$, $p<0.05$) as was the three-way interaction between subject, tooth class and tooth aspect ($F=2.7$, $R^2=0.035$, $p < 0.05$). Interpretation of these

interactions is provided in the main manuscript.

Though it is possible to interpret the magnitude of the effect sizes in an analysis of non-independent data, we caution against interpreting the p-values associated with the Adonis model since the existence of a spatial series, as revealed in subsequent analyses, implies that samples within a mouth are not independent of one another. Violation of the assumption of non-independence of samples increases the likelihood that the null hypothesis, that there is no effect for a given factor, will be rejected which makes interpreting p-values tricky.

Interpretation of other PCoA axes

A screeplot (Supplementary Data 3) of the PCoA presented in Figure 1 suggested that it would be wise to examine the first, second, third and fourth principal coordinates, which were plotted in all pairwise combinations (Supplementary Figure 1). Molars and incisors appeared to separate from one another, particularly at the lingual aspect of teeth, along axis 1 but not along axis 2, axis 3 or axis 4, suggesting that axis 1 scores may be an appropriate focal piece for follow up analysis. Further, inspection of the ordination plots involving axis 1 revealed the absence of any "horseshoe" or parabola shape that might result in the confounding of sample distributions along axis 1.

Investigation of the impact of data transformation on primary findings

To evaluate the impact that the choice of data transformation method had on the observed difference between the molars and incisors, we next performed a PCoA on the Euclidean distance between samples after applying various transformations to the species x sample data

matrix (Supplementary Figure 2). The molar and incisor samples clearly separated from each other along axis 1 when data were subject to the Chord and Hellinger data transformations. Samples did not separate as much by tooth class when the VST-transformed species abundance profiles were directly ordinated with the Euclidean distance, but molars and incisor samples did separate by tooth class following computation of Bray Curtis dissimilarity on VST-transformed or relative-abundance transformed data.

Investigation of the impact of beta diversity metric on primary findings

Next, we sought to evaluate the robustness of the separation of the molars/incisors to a variety of distance metrics (Supplementary Figure 3). Regardless of the chosen metric (Binomial, Bray, Canberra, Euclidean, Gower, Jaccard, Kulczynksi, and Manhattan), molars and incisors separated along axis 1, particularly when samples were collected from the lingual aspect of teeth.

Taken together, these results suggest that the separation of communities inhabiting the molars and incisors is robust to a variety of different transformations and distance metrics.

Supplementary Note 2. Temporal stability of oral microbial communities

Biogeographic surveys often address the extent to which communities change over time. For microbial communities of the oral cavity, the interaction between space and time has been inadequately addressed, particularly for communities of dental plaque. For this reason, we sought to evaluate the extent to which supragingival sites that were sampled on a daily basis vary over the course of a month within individuals.

Analyzing a multivariate generalization of the correlation coefficient over increasing time intervals revealed that the similarity between communities decreased as a function of increasing time interval (Supplementary Figure 4). All within-individual rates of change were modest, negative and significant (range of slopes: -0.006, -0.002, $p < 0.05$) which suggests that community similarity gradually decayed over the course of a month. This gradual decay in community similarity reflects random temporal fluctuations in the abundances of individual ASVs, an observation consistent with previous reports[3].

When RV coefficients were independently computed for each plaque habitat, molar communities were found to share significantly higher RV coefficients than incisor communities irrespective of tooth aspect or jaw (Supplementary Data 2). The higher RV coefficients associated with molar communities suggests molar sites are more stable than incisor sites. The trend towards higher temporal variation at the incisors compared to the molars likely reflects the influence of salivary flow, oral hygiene and abrasion due to the tongue.

One question regarding temporal variation that was not addressed by our study is whether soft-tissue communities such as those inhabiting the buccal mucosa change over time at rates that are similar to or less than those of supragingival sites. Given that the forces that shape turnover (e.g., the presence or absence of desquamation and invasion of epithelial cells, etc.) at oral and hard tissue habitats differ, differences in patterns of temporal stability at each of these sites might be considerable. Quantification of the differences between communities inhabiting these diverse sites may provide insight into the ecology of the communities of the human oral cavity and their relationship to health and disease.

Supplementary Note 3. Communities vary along an anterior-posterior gradient - extended spatial analysis

As part of the trend surface analysis (Figure 2), we performed forward selection of the polynomial terms. The complete model which incorporated all 9 polynomial terms explained 3.59% of the total variance, while the reduced model, which included only 6 polynomial terms, explained 3.25%.

A permutational ANOVA was performed to identify the RDA axes of the reduced model that corresponded to significant linearly independent spatial structures [4]. The first RDA axes appeared to distinguish between tooth class in a manner consistent with the previously described community gradient (Figure 2). By projecting RDA1 scores onto a map of the mouth, another difference emerged: maxillary lingual sites differed from maxillary buccal sites, mandibular buccal, and mandibular lingual sites (Supplementary Figure 7). Strikingly, the third RDA axis appeared to mirror the first, highlighting the difference between the lingual and buccal tooth aspects in the mandible. The arrangement of the first and third RDA axes suggests that differences in community composition vary by tooth aspect and jaw. Of special interest, the second and fourth RDA axes highlighted differences between the anterior and posterior mouth. In addition, they appeared to reveal an interaction between oral compartment (anterior/posterior) and tooth aspect (buccal/lingual) since buccal sites distinctly differed from lingual sites although to a lesser degree than variation along the anterior-posterior dimension.

Of 9 polynomial terms, 6 were found to be significant. The variable X^2 separated anterior from

posterior teeth, a pattern that differed by tooth aspect (buccal/lingual) as evidenced by the positive and negative scores for the buccal and lingual molars, respectively (Supplementary Figure 7). Y^2 similarly distinguished between anterior and posterior teeth but appeared to weight the buccal and lingual surfaces in the anterior mouth differently, while sites in the posterior were invariant with respect to scores along this term. Y distinguished between the upper and lower jaws while Y^3 distinguished not only between anterior and posterior teeth, but also the buccal and lingual surfaces of teeth in the anterior mouth. X^2Y differently weighted tooth aspect but only at the posterior teeth, and not at anterior teeth, irrespective of jaw. X^3 seemed to differentially weight the left and right sides of the mouth, but only for lingual and not buccal tooth surfaces.

Taken together, these data suggested that not only do communities vary from front to back of the mouth, but that tooth aspect and jaw are also associated with differences in community composition.

Supplementary Note 4. PCNM

A common complaint about trend surface analysis is that it is only able to highlight broad scale spatial patterns. On the other hand, Principal Components Analysis of Neighbor Matrices (PCNM) allows for the detection of both broad and fine scale spatial patterns[4]. PCNM variables are generated by taking the eigenvectors of a principal coordinates analysis on a neighbor matrix, which is generated by defining as neighbors any site within an arbitrarily defined distance threshold (often the minimal distance that gives rise to a connected network graph). As a consequence, the distance threshold used to define the network will determine which PCNM

variables are generated and at what spatial scales (e.g., broad to fine scale) they occur. These eigenvectors (i.e., the PCNM variables) are then used as spatial predictors in a multivariate analog of linear regression on the species by sample data matrix using RDA. As a consequence, each RDA yields ordination axes that maximize the variance in community composition across a linear combination of the spatial predictors, the PCNM variables. Permutation testing for significant RDA axes allows one to interpret significant RDA axes as corresponding to significant spatial structures.

To examine fine scale spatial patterns in community composition, we next performed a PCNM analysis of our species x sample data matrix, defining as a neighborhood the minimal Euclidean distance that gave rise to a connected network. As with the trend surface analysis, the PCNM variables used as constraints in the spatial model were subject to forward selection. The adjusted R^2 of the global model which included all 24 PCNM variables was 0.0271, while the R^2 of the adjusted minimum model was 0.0268 after retention of just 8 significant PCNM variables (V4, V20, V17, V23, V18, V5, V14, and V6).

Permutation testing identified 3 significant RDA axes from the PCNM analysis that aligned with spatial structures. As might be expected from the trend surface analysis, the first RDA axis separated anterior from posterior teeth irrespective of jaw or tooth aspect (Supplementary Figure 8a). Of the significant PCNM variables, V4 and V5 seemed to align with the first RDA axis (Supplementary Figure 8b). The second and third RDA axes corresponded to finer scale spatial structures – the second axis may partially explain differences between tooth aspect (buccal, lingual) since at least in the case of 19 (out of 30) teeth, axis 2 scores were negative at one tooth

aspect and positive at the other tooth aspect.

The third axis was difficult to interpret. The bulk of the remaining PCNM variables (V20, V17, V23, V18, V14, V6) were also difficult to interpret – an observation we attempt to address using model selection. What is clear from this PCNM analysis is that the first axis highlights the difference across the anterior to posterior dimension, a pattern that was observed with the trend surface analysis.

Supplementary Note 5. Model Selection and Moran's Eigenvector Maps (MEM)

The PCNM and in particular, the difficulties inherent in interpreting some of the finer-scale structures described by the PCNM variables raised a question about how a neighborhood should be defined. The arbitrary cutoff that was used in the PCNM dictated the structure of the PCNM variables by setting the neighborhood size at an arbitrary point (e.g., by defining what sites are near vs. far). One way to avoid using an arbitrary cutoff is to construct a constellation of different models each differing in the definition of a neighbor that is used to construct the PCNM variables. After generating a variety of different models, the best model can be selected by identifying one that maximizes variance and/or minimizes some model selection feature like the Akaike information criterion (AIC). Towards this end, we constructed a set of 20 neighborhood matrices with thresholds that varied between 0.2 and 2; thus, some models had thresholds lower than that used in the first PCNM (Supplementary Figure 8) and some models had thresholds that were much higher than that used in the first PCNM.

We compared the set of 20 models, and the neighborhood matrix in which AIC was minimized had 55 MEM variables defined at a threshold value of 1.04, nearly twice that of the arbitrary threshold used in the first PCNM. This suggests that the common practice of using a minimal spanning tree to identify the distance threshold that defines a neighborhood may not be ideal when it comes to generating optimal spatial predictors of community composition in the context of the human oral cavity.

We next analyzed the RDA axes that were identified by permutation as significant: RDA1 corresponded to the spatial gradient across the anterior-posterior dimension, particularly differentiating sites at the lingual surfaces of teeth. RDA2 distinguished between anterior and posterior sites in the lower jaw; in the upper jaw, by contrast, RDA2 distinguished between the buccal and lingual tooth aspects. The third axis seemed to strongly correspond to the differentiation of anterior vs. posterior sites in both jaws and at both tooth aspects.

Forward selection on the 55 MEM variables revealed that 20 PCNM variables significantly contributed to the observed variation (Supplementary Fig 9b). The MEM variable (X4) that explained the greatest amount of the variation in the data was one that distinguished between anterior and posterior sites irrespective of jaw (Supplementary Figure 9b; Supplementary Data 4). The next most powerful MEM variable (X6) distinguished between buccal and lingual sites in the maxilla. The MEM variable that explained the least amount of variation in the data (X55) was similar to X4 in that it differentiated anterior and posterior sites.

The fine-scale PCNM variables appeared to be easier to interpret in the MEM model (Supplementary Figure 9b) when compared to the first PCNM model (Supplementary Figure 8b) that was generated with an arbitrary threshold. Of the 20 significant MEM variables, 7 (X27, X29, X31, X32, X34, X35, X36) corresponded to fine scale spatial patterns (Supplementary Figure 9b). Notably, most of the fine scale spatial variables identified by the MEM model mapped to anterior sites, suggesting that each anterior tooth is colonized by a unique microbial community that is distinctive from neighboring teeth.

The observation that the anterior to posterior gradient emerged as a significant pattern in all analyzed spatial models (trend surface analysis, PCNM, MEM) suggested that the gradient is unlikely to be an artifact, but rather a true biological signal.

Supplementary Note 6. Extended spatial analysis of the mucosal biogeography data

Since only the first RDA axis was interpreted in the main manuscript, we present an extended spatial analysis for the mucosal biogeography data here, providing an interpretation of other significant ordination axes and additionally assessing the extent to which different polynomial terms explained the significant spatial patterns in community composition.

The complete trend surface model, which included 9 polynomial terms, explained 7.54% of the total variance while the reduced model, which retained just 5 polynomial terms, explained 7.58%. The variable X^2 appeared to separate anterior from posterior sites, a pattern that seemed to differ between buccal and lingual posterior (but not anterior) surfaces, as evidenced by the

opposing scores for the buccal and lingual molars (Supplementary Data 3). Y^2 and Y^3 similarly distinguished between anterior and posterior teeth but they also appeared to differently weight the buccal and lingual surfaces. Y, on the other hand, differentiated communities based on whether they were located in the upper vs. the lower jaw. X^2Y differentiated buccal and lingual posterior teeth irrespective of jaw, while X differentially weighted the left and right sides of the mouth, but only for lingual and not for buccal tooth surfaces.

As mentioned previously, the significant RDA axes can be interpreted as linearly independent spatial structures[4]. The first RDA axis appeared to distinguish between tooth classes in a manner consistent with an ecological gradient (Figure 3). The second axis appeared to separate sites based on jaw while the third axis also aligned with differences across the anterior-posterior dimension (Supplementary Figure 10).

Supplementary Note 7. Clinical variables that distinguish the low flow and control cohorts

As expected based on previous research[5], the mean unstimulated whole salivary flow rate (UWS-FR) of the low-flow, Sjögren's Syndrome (SS) patients enrolled in this study (mean, 0.09 mL/min) was significantly less than that of the controls (0.51 mL/min; Welch's T-Test, $p < 0.05$). SWS-FR correlated with UWS-FR (R^2=0.66) and was also significantly lower in the SS cohort compared to controls (t=5.00, df=11.2, $p < 0.05$).

Though none of the participants in the validation cohort had active dental caries at the time of sample collection, the number of missing and filled smooth surfaces (MFS) differed between the

low flow and control cohorts. Only 2 of 7 individuals in the control cohort had smooth surface MFS and they were limited to the molars and premolars. On the other hand, 9 of 10 individuals in the low flow cohort had smooth surface MFS on molars and premolars, with 40% of low-flow individuals showing past evidence of decay on the smooth surfaces of the incisors and/or canines, as well (Supplementary Figure 12).

Both healthy individuals and individuals with low salivary flow tend to experience caries on the biting surfaces of posterior teeth, which have anatomically normal pits and fissures that serve as habitats for cariogenic bacteria. In addition, individuals with low salivary flow experience an atypically high rate of caries on teeth in the front of the mouth, particularly on roots and other smooth surfaces. Generally, these smooth surfaces are thought to be protected from caries by the ease with which we can brush these surfaces; by the buffering activity of saliva; and by the rapid rate of oral clearance from the anterior compartment.

In this work, we found an enrichment of caries-associated bacteria in patients with low rates of salivary flow. At the same time, we report that the community gradient across the anterior-posterior dimension appears to be modified (e.g., attenuated) in most of these patients with low salivary flow. One (unanswered) question raised by these patterns is whether the community gradient is also modified in individuals with a history of extensive caries due to reasons other than reduced salivary flow – that is, whether any disturbance that decreases or alters the landscape heterogeneity of the oral ecosystem will result in similar findings.

Supplementary Note 8. Community pattern can be described as a nested subset with clumped species loss

Metacommunity theory encompasses six idealized patterns that describe the distribution of communities across geographic space, including: 1) nested subsets; 2) checkerboards; 3) Clementsian gradients; 4) Gleasonian gradients; 5) evenly spaced gradients; and 6) random distributions[6]. Each idealized pattern describes the variation of species among sites when a principal axis distinguishes communities along a major gradient. Importantly, if species are not structured along a common gradient, then the metacommunity will not exhibit coherence[7]. Nonetheless, communities that lack coherence may respond differently to multiple different gradients or they may exhibit the checkerboard pattern. The elements of metacommunity structure (EMS) approach, like many other spatial models, is concerned with identifying spatial patterns and cannot identify the processes that give rise to the spatial pattern.

We sought to identify which of the idealized patterns best describes the anterior-posterior gradient using the EMS perspective. For this reason, we computed community coherence, turnover and boundary clumping, the three metrics that can be used to differentiate the six idealized patterns.

We first evaluated coherence, a metric that assesses the degree to which species ranges overlap across sites structured along a gradient. Coherence can be evaluated using an incidence matrix in which site occupancy by a given species is denoted by a ‘1’ and absence at a site by that species is denoted by ‘0’. A species range is “completely coherent” if the species occupies all sites without any absences across the range. For example, in a scenario where there are 5 sites and one

species, a “completely coherent” range would be defined as [1, 1, 1, 1, 1] since the single species occupies all sites. Other patterns of coherence may appear as [0, 1, 1, 1, 1] or [0, 1, 1, 1, 0] or [0, 1, 1, 0, 0, 0] and so on, encompassing any variant in which the sequence of ‘1’s’ (i.e., site occupancy) continues without being interrupted by an absence (i.e., 0). Interruptions in site occupancy by an absence such as is the case in the set [0, 1, 1, 0, 1] are defined as an ‘embedded absence’. As the number of embedded absences increases, the degree of coherence decreases. The observed number of embedded absences can be compared against the mean of a null model, generated by simulating random matrices, in order to evaluate significance.

In the case of a community, the species x sample data matrix is ordered by the EMS framework in a way that maximizes the coherence of all species across the set of sites. ‘Negative coherence’ is defined by situations in which the number of embedded absences significantly exceeds the number expected by random chance. A significantly higher than expected number of embedded absences (i.e., negative coherence) suggests that communities conform to a ‘checkerboard pattern’. Checkerboard patterns occur when pairs of species have ranges that exclude each other at sites across their ranges. On the other hand, positive coherence is defined as situations where the number of embedded absences is significantly less than that suggested by the random model.

Regardless of the subject’s health status, coherence as measured by the number of embedded absences was significant (Supplementary Data 5; Supplementary Figure 14a; Supplementary Figure 14d). Moreover, the number of embedded absences was, in all cases, less than the expected number of embedded absences (Supplementary Figure 14a; Supplementary Figure 14d). This is consistent with a pattern of positive coherence, allowing us to rule out the

possibility that the spatial pattern across the anterior-posterior dimension corresponds to either a checkerboard pattern or a null pattern. In fact, positive coherence implies that species are structured along at least one common latent gradient.

EMS theory suggests that inspection of community turnover and boundary clumping can yield additional insight into metacommunities that have fewer embedded absences than expected by chance[7]. For this reason, we next computed turnover for each subject enrolled in the study (Supplementary Data 5; Supplementary Figure 14b; Supplementary Figure 14e). In all cases, turnover (i.e., the number of times that a species was observed to replace another when moving from one site to the next site) was significantly less than expected based on the random model. Such a pattern – one in which species have coherent ranges but do not replace one another when moving from one site to the next – corresponds to a nested subset model. Put another way, communities exhibiting a nested structure tend to experience less turnover than expected along environmental gradients.

The pattern of species loss across the gradient can be investigated by examining 'boundary clumping', a metric that assesses the extent to which the boundaries of different species ranges cluster. In the case of nested subsets, species loss can occur in a clumped manner, a random manner, or a hyper-dispersed manner[7]. Upon examination of our data, we observed that in all cases the Morisita index, used to estimate the boundaries of ranges, exceeded 1, indicating that the boundaries of species ranges tend to be more clumped than expected by chance (Supplementary Figure 14e). This suggests that communities are distinct (i.e., Clementsian) but that groups of species are lost at only one end of the gradient, perhaps in the anterior

compartment where species richness tended to be less (Supplementary Data 3). Taken together, these results imply that the type of spatial gradient can best be characterized as a nested subset with clumped species loss across the gradient.

Using multivariate methods in spatial ecology (Trend Surface Analysis, PCNM, MEM), we show that an ecological gradient gives rise to a gradient in community composition that distinguishes between sites in the anterior and posterior portions of the mouth. Since this gradient appears to be modulated in patients with low salivary flow, we hypothesized that salivary flow plays a role in structuring the gradient through the generation of a pH differential between sites. Analysis of EMS provided further insight, suggesting the gradient in community composition reflects differential abundance as well as gradients in species richness and species incidence across the anterior-posterior dimension.

The detection of these three types of gradients in community composition emphasizes the interplay between an underlying environmental gradient and the lifestyle preferences of individual species[8], rather than providing support for a checkerboard pattern. The movement of salivary flow through the mouth, the brushing habits of the human host, and/or abrasion due to the movement of the tongue may each restrict alpha diversity and species incidence at anterior sites, giving rise to the patterns observed here.

Though we did not quantify group differences, we observed that communities in the mouths of individuals who experienced low salivary flow due to SS also experienced less turnover and more clumped boundaries compared to healthy controls. This may suggest that as salivary flow decreases, the underlying environmental gradient becomes attenuated, resulting in the

homogenization of intra-oral sites and the attenuation of the gradient in community composition, species richness and species incidence.

Supplementary Methods

Sample collection sites and protocol

Most tooth specimens were collected using Foam Catch-All Collection Swabs (Epicentre #QEC091H). Alternatively, as indicated in the sample mapping files (Supplementary Data 6), a subset of sites for two subjects in the discovery data set (P2-1 and P2-2) were sampled using the Bristle Catch-All Collection Swabs (Epicentre #MB100BR).

Swabs were applied to each surface in a circular motion with moderate pressure for 5-20 seconds. Samples collected by subjects were temporarily stored in home freezers at -20°C until transported on ice to UCSF on Days 1, 8, 15, 22, and/or 29.

Subject-collected specimens were subsequently placed on dry ice for transport to Stanford where they were stored at -80°C until DNA extraction. All clinician-collected specimens were immediately frozen on dry ice for transport from UCSF to Stanford where the samples were stored at -80°C.

Demultiplex and quality filtering – validation and biogeography data sets

A total of 2.35×10^9 raw reads were analyzed. Forward and reverse reads were independently de-multiplexed using the *split_libraries_fastq.py* command in Qiime[9] with parameters tuned to

prevent quality filtering. Sequences were parsed into sample-specific files using the *split_sequence_file_on_sample_ids.py* command in Qiime before import into R-3.3.0 for quality filtering with the R package dada2[10]; the first 10 nucleotides (5') of each read were trimmed followed by the truncation of forward and reverse reads at lengths of 240 and 160 nucleotides, respectively. Reads were eliminated if the maximum expected error exceeded 2; reads were also truncated at the first instance in the sequence where the quality score was less than 2. Sequences were de-replicated before inference of sequence-specific errors and elimination of problematic reads. Dereplicated and filtered forward sequences were subsequently merged with their paired-end reads before construction of an amplicon sequence variant (ASV) table.

The removeBimeraDenovo function of dada2 set to the 'consensus' method was used to filter chimeras from each ASV table. Taxonomic assignment was then performed down to the species level where possible using the dada2 implementation of the RDP Naive Bayesian Classifier trained on the RDP database (version 14.0)[11]. Sequences were subsequently subjected to mega-blast against the 16S rRNA NCBI database and additional taxonomic assignments were made if only a hit matched a single query species in the database with a similarity of ≥98%.

For the validation data set, the preceding quality filtering steps were performed independently for each of 15 sequencing runs, generating 15 independent ASV tables which were merged using dada2 . After merging all data in the validation data set, the unfiltered ASV table consisted of 28,603 taxa across 7,446 samples and encompassed 545,864,731 sequences.

The unfiltered mucosal biogeography data set, which was analyzed independently, consisted of

3,133 taxa distributed across 701 samples and encompassed 52,743,939 high-quality reads.

Decontaminating the ASV table

To identify taxa in the validation data set associated with contamination, a data subset was generated consisting only of the technical controls (N=431), which were sequenced in parallel with true samples (N=7,015) and the mean abundance of each taxon was computed across all control samples. Similarly, a data subset consisting only of true samples was generated, and the mean abundance of each taxon in the true samples was computed. The mean abundance in true samples was centered by subtracting, for each taxon, the mean abundance in controls. This resulted in negative observed values for 5,487 taxa, indicating that these taxa were more abundant in the controls than they were in the true samples. Scrutiny of the taxonomic profiles of these taxa indicated that they were unlikely to have originated from the oral cavity, and they were consequently dropped from the validation data set.

Second, the sample enrichment score for each taxon was calculated by dividing the prevalence of each taxon in samples by the prevalence of each taxon in controls. Taxa were dropped if they were enriched in controls compared to samples (i.e., if the prevalence was higher in controls than in samples). While this second approach eliminated taxa that were unlikely to have originated from true samples of the human oral cavity, it may have also eliminated taxa that were likely to have been cross-contaminants in the controls because of high prevalence/abundance in the oral samples. Filtering using this method (Supplementary Data 1) reduced the data table for the validation data set to just 480 taxa across 7,002 samples.

Calculation of missing, filled surfaces (MFS)

The missing, filled surfaces (MFS) index was calculated based on the number of missing and filled surfaces at either the buccal or lingual aspect of tooth smooth surfaces.

Separation of molars and incisors is robust to a variety of data transformations

To evaluate the robustness of the separation of molars and incisors to a variety of different transformations, the raw data table was subjected to the following transformations: Chord, Hellinger, relative-abundance, chi-square, and variance-stabilizing transformation (VST), prior to principal coordinates analysis (PCoA) of the Euclidean distance between samples or in the case of VST-transformed and relative-abundance-transformed data before PCoA of the Bray Curtis distance metric. ggplot2 was used to visualize sample scores projected onto the first and second coordinates of each PCoA.

Separation of molars and incisors is robust to a variety of distance metrics

The robustness of the separation of molars and incisors to a variety of distance metrics was evaluated by confirmatory ordinations using the non-phylogenetic distance metrics available in vegan. The decostand function was used to convert absolute counts to relative abundances before the computation of the following distance metrics: Euclidean, Manhattan, Canberra, Bray Curtis, Kulczynski, Jaccard, Gower, and Binomial. A subset of distance metrics was computed on raw abundance values, including altGower, Morisita, horn, and Rao. The plot_ordination function in phyloseq[12] was used to visualize all PCoA ordinations.

Forward selection – trend surface analysis

The poly function in base R was used to generate an orthogonal 3rd degree polynomial function of the geographic coordinates of sample sites. A redundancy analysis (RDA) was performed on the Hellinger transformed data and the polynomial terms were used as the constraint. The RsquareAdj function of the vegan package was used to obtain the R^2 value for the full model. The R^2 of the complete model was set as the stopping value for the forward selection of significant polynomial terms as evaluated with the forward.sel function of the packfor package[13]. An independent RDA was performed with the significant polynomial terms. The overall model and the RDA axes were independently tested for significance using anova.cca function of vegan. The significant RDA axes and polynomial terms were subsequently visualized using either ggplot2 or the s.value function of the ade4 package.

Principal coordinates of neighbor matrices (PCNM)

The quickPCNM function of the PCNM package[14] was used with default settings to truncate the Euclidean distance at 0.5763064, generating 24 PCNM eigenvectors. A significant linear trend was found in the data which were then detrended using the built-in quickPCNM function. The R^2 of the global model was 0.0271 and the R^2 of the minimum model in which only 8 PCNM variables were retained, following forward selection, was 0.0268. Permutational analysis of the RDA under the reduced model revealed 3 significant RDA axes. The set of significant RDA axes and PCNM variables were visualized by ggplot2.

Model selection using Moran's Eigenvector Maps (MEM)

In addition to performing a trend surface analysis, we also performed a principal coordinates

analysis of neighbor matrices (PCNM), which is suited for analyzing spatial structure at various scales ranging from the broadest scale such as what is captured in the trend surface analysis to a scale that is finer than what can be analyzed using trend surface analysis[4]. The neighborhood graph (Supplementary Data 4) was constructed using the dnearneigh function of the spdep package: a set of 20 different models was created each differing along an evenly spaced distance between 0.2 and 2 (ref[15]). The distance between nearest neighbors was computed using the nbdists function of the spdep package. The spatial weights were defined as a function of the distance between neighbors, and the spatial weight matrix was created using the nb2listw function. The scores.listw function of the spacemakeR package[16]was used to compute eigenvectors of the centered matrix of spatial weights. The centered spatial weights were subsequently used as predictors of variation in a Hellinger-transformed species x sample data matrix and the performance of the models was assessed using the ortho.AIC function of spacemaker. The best model was determined to be the one that minimized the *Akaike information criterion* (AIC). The eigenvectors were extracted from the model along with significant RDA axes, which were visualized using ggplot2.

Assessing the role of sequencing depth and alpha diversity

To determine if sequencing depth could explain the gradient in community composition, we performed a Wilcoxon rank sum test on the difference in sequencing depth between molars and incisors for each tooth aspect. In addition, we tested the significance of differences in alpha diversity (Chao1, Shannon and Simpson indices) between the molars and incisors using a Wilcoxon rank sum test.

Assessing the stability of the gradient at varying abundance thresholds

In order to determine the sensitivity of the gradient shown in Figure 2 to different taxonomic abundance thresholds, we performed independent trend surface analyses on several data subsets defined by thresholding the data set, including only the top-most abundant taxa at each threshold: 10, 20, 30, 40, 50, 60, 70, 80, 90, and 100. Sample scores on the first principal coordinate (y-axis) were subsequently plotted against tooth number (x-axis) using the package ggplot2 (ref[17]).

Identifying taxa that exhibit spatial dependence in count distributions

A between-taxa correlation matrix was generated for the 70-most abundant taxa using the ggcorplot package[18]. To perform an explicit test of spatial dependence for these 70 taxa, we computed Moran's *I* Coefficient[14] for spatial autocorrelation. The physical, 2-dimensional distance separating sites was computed as the inverse Euclidean distance using the vegdist function of the vegan package. The inverse Euclidean distance was then used as input for the sdpep package[19] which was used to generate the spatial weights. To eliminate the violation of non-independence of temporal samples in a spatial series, all samples belonging to a single site (2B, 2L, 3B, 3L, 4B ... 30B, 30L) were merged by summing each ASV within each site.

We tested the null hypothesis that ASVs vary across teeth in a manner independent of the physical distance between sample sites. Significance was assessed after a Bonferroni correction on simulated data generated by Monte Carlo simulations (N=1,000). A Moran's *I* coefficient was determined to be significant if it could be distinguished from zero (Bonferroni corrected $p < 0.05$). To contextualize the pattern of spatial dependence among these spatially variant taxa, taxa counts were visualized as a function of tooth number using ggplot2 (Figure 2c, Supplementary

Data 2). Select taxa were displayed in Figure 2a because they were moderately abundant and appeared to follow the same distribution across multiple subjects.

Examining the relationship of oral biogeography to tissue type

To examine the overall structure of the data, we used the phyloseq function plot_ordination to visualize a PCoA on Bray Curtis dissimilarity using the mucosal biogeography data set as the input. Sample scores were plotted against the first and second axes and samples were colored based on whether they originated from a hard (supragingival) or soft (alveolar mucosa, keratinized gingiva, buccal mucosa) surface.

Examining the temporal stability of oral microbial communities

A k-tables analysis[20] was used to examine the temporal variation of microbial communities on a site-by-site basis (molars, incisors) within each of 8 subjects (90 taxa, 3393 samples). A variance stabilizing transformation was used on taxa counts before the statis function of ade4 was applied to a list of ASV tables, one defined for each day (20-28 days). The RV coefficient[21] which can be interpreted as a multivariate generalization of a correlation coefficient was plotted as a function of time to examine whether the correlation amongst samples was stable or whether they experienced gradual decay over time. To assess the relative stability of molar compared to incisor communities, the RV coefficient was separately computed for each habitat (e.g., 3 buccal, 3 lingual, 4 buccal, 4 lingual, etc.) and significance was evaluated using the Wilcoxon rank sum test.

Metacommunity analysis using the metacom package

To assess the type of spatial pattern that best described the anterior-posterior gradient, we used the framework developed by Leibold & Mikkelson (2002) as implemented in R[22] to assess coherence, turnover, and boundary clumping.

First, coherence was computed using the Coherence function based on an ordered ordination of the presence-absence matrix with the “r1” method. The number of ‘embedded absences’ in each matrix was determined by comparison to the simulated mean of 1000 random matrices. Coherence was computed on the presence-absence matrix for the following subjects (1-101, 1-102, 1-107, 3-301, 3-302, 3-303, 3-304, 3-305, 3-306, 3-307). In addition, due to computational limitations, since we could not generate coherence estimates for all healthy control subjects, the number of taxa at each sample site was summed across subjects and coherence was computed on the “healthy control representative table” as well as on the “low flow representative table”.

Since coherence indicated that the number of embedded absences could be distinguished from the null model, we next computed turnover for each data matrix independently using the Turnover function of the metacom package with the “r1” method and 1000 simulations. In addition, boundary clumping was computed with the BoundaryClump function of the metacom package.

Coherence, turnover and boundary clumping were plotted as a function of the subject's health status using the ggplot2 package. In addition, Coherence and turnover estimates were plotted against their simulated means using ggplot2.

Supplementary References

1. Torlakovic L, Klepac-Ceraj V, Ogaard B, Cotton SL, Paster BJ, Olsen I. Microbial community succession on developing lesions on human enamel. J Oral Microbiol **4**, 16125 (2012).

2. Mark Welch JL, Rossetti BJ, Rieken CW, Dewhirst FE, Borisy GG. Biogeography of a human oral microbiome at the micron scale. *Proc Natl Acad Sci U S A* **113**, E791-800 (2016).

3. Utter DR, Mark Welch JL, Borisy GG. Individuality, Stability, and Variability of the Plaque Microbiome. *Front Microbiol* **7**, 564 (2016).

4. Borcard D, Gillet F, Legendre P. *Numerical Ecology with R*. Springer (2011).

5. Pijpe J, Kalk WW, Bootsma H, Spijkervet FK, Kallenberg CG, Vissink A. Progression of salivary gland dysfunction in patients with Sjogren's syndrome. *Annals of the rheumatic diseases* **66**, 107-112 (2007).

6. Leibold MA, Mikkelson GM. Coherence, species turnover, and boundary clumping: elements of meta-community structure. *OIKOS* **97**, 237-250 (2002).

7. Presley SJ, Higgins CL, Willig MR. A comprehensive framework for the evaluation of metacommunity structure. *OIKOS* **119**, 908-917 (2010).

8. Ulrich W, Almeida-Neto M. On the meanings of nestedness: back to the basics. *Ecography* **35**, 865-871 (2012).

9. Caporaso JG*, et al.* QIIME allows analysis of high-throughput community sequencing data. *Nat Methods* **7**, 335-336 (2010).

10. Callahan BJ, McMurdie PJ, Rosen MJ, Han AW, Johnson AJ, Holmes SP. DADA2: High-resolution sample inference from Illumina amplicon data. *Nat Methods* **13**, 581-583 (2016).

11. Cole JR*, et al.* The Ribosomal Database Project (RDP-II): sequences and tools for high-throughput rRNA analysis. *Nucleic acids research* **33**, D294-296 (2005).

12. McMurdie PJ, Holmes S. Phyloseq: a bioconductor package for handling and analysis of high-throughput phylogenetic sequence data. Pacific Symposium on Biocomputing **2012**, 235-246 (2011).

13. Dray S, Legendre P, Blanchet G. packfor: Forward Selection with permutation. https://rdrr.io/rforge/packfor/. (2016).

14. Dray S, Legendre P, Peres-Neto PR. Spatial modelling: a comprehensive framework for principal coordinate analysis of neighbour matrices (PCNM). *Ecological Modelling* **196**, 483-493 (2006).

15. Bivand R, Piras G. Comparing implementations of estimation methods for spatial econometrics. *Journal of Statistical Software* **63**, 1-36 (2015).

16. Dray S. spacemakeR: Spatial modelling. https://rdrr.io/rforge/spacemakeR/. (2013).

17. Wickham H. *ggplot2: Elegant Graphics for Data Analysis*. Springer (2009).

18. Kassambara A. ggcorrplot: Visualization of a correlation matrix using 'ggplot2'. https://cran.r-project.org/web/packages/ggcorrplot/index.html. (2016).

19. Dray S, Jombart T. Revisiting Guerry's data: Introducing spatial constraints in multivariate analysis. *Annals of Applied Statistics* **5**, 2278-2299 (2011).

20. Dray S, Chessel D, Thioulouse J. Co-inertia analysis and the linking of ecological data tables. *Ecology* **84**, 3078-3089 (2003).

21. Robert P, Escoufier Y. Unifying tool for linear multivariate statistical-methods - RV-Coefficient. *Journal of the Royal Statistical Society Series C-Applied Statistics* **25**, 257-265 (1976).

22. Dallas T. metacom: an R package for the analysis of meta- community structure. *Ecography* **37**, 402-405 (2014).
