# Supplementary material for: A spatial gradient of bacterial diversity in the human oral cavity shaped by salivary flow
Source: Nat Commun. 2018 Feb 14;9:681. doi: 10.1038/s41467-018-02900-1 (PMC5813034; doi:10.1038/s41467-018-02900-1)
Supplement: Supplementary file 3 — Description of Additional Supplementary Files [file 41467_2018_2900_MOESM3_ESM.pdf]

Supplementary Data

Supplementary data 1

Supplementary data 1 contains the code used to decontaminate the validation dataset.

Supplementary data 2

Supplementary data 2 contains all the code needed to reproduce most of the supplementary figures excluding those associated with the Principal Coordinates of Neighbor Matrices (PCNM), the Moran's Eigenvector Map's (MEM) or the Elements of Metacommunity Structure (EMS). Supplementary data 2 also includes the code needed to reproduce the findings for the discovery dataset.

Supplementary data 3

Supplementary data 3 contains all the code needed to reproduce the figures in the main manuscript.

Supplementary data file 4

Supplementary data 4 contains all the code needed to perform the PCNM and the MEM analyses.

Supplementary data file 5

Supplementary data 5 contains all the code needed to reproduce the results of the EMS analysis.

Supplementary data 6

Supplementary data 6 is an Excel workbook that contains 4 data sets:

1) ASV table, taxonomy file, and sample data mapping file for the discovery data set;
2) ASV table, taxonomy file, and sample data mapping file for the decontaminated data set;
3) ASV table, taxonomy file, and sample data mapping file for the mucosal biogeography data set;
4) ASV table, taxonomy file, and sample data mapping file for the validation data set.
